# An illustrated key to the fiddler crabs (Crustacea, Decapoda, Ocypodidae) from the Atlantic coast of Brazil

**DOI:** 10.3897/zookeys.943.52773

**Published:** 2020-06-22

**Authors:** Setuko Masunari, Salise Brandt Martins, André Fernando Miyadi Anacleto

**Affiliations:** 1 Laboratory for Crustacean Research UFPR, Department of Zoology, Federal University of Paraná, Curitiba, Paraná State, Brazil Federal University of Paraná Curitiba Brazil

**Keywords:** Biological notes, distribution, mangrove, recognition characters, tidal flats

## Abstract

Fiddler crabs are one of the most notable animal groups in Brazilian estuarine environments, due to their high density and characteristic waving of males. An illustrated key to the ten species recorded as far in the country is provided using only clearly visible characters of males. Furthermore, additional recognition characters, information about geographic distribution and biology of each species are presented. Most examined crabs were collected in Guaratuba Bay, southern Brazil.

## Introduction

Studies on fiddler crabs began more than 300 years ago, certainly because the observers were attracted to the immense claw of males that were tirelessly waving in a typical movement and rhythm. Fiddler crabs are semi-terrestrial decapod crustaceans and inhabit shaded substrates of mangrove forest or sunny tidal flats adjacent to it. During high tides they hide in individual burrows that are the center of a fiddler crab life (Crane 1975).

Ten species of fiddler crabs are known from the Brazilian Atlantic coast (Melo 1996), with the newest species described more than 30 years ago by von Hagen (1987). Brazilian population studies, however, began only at the beginning of the present century, and a great deal of knowledge was generated since then (see partial review in Ribeiro and Bezerra 2014).

Despite the valuable identification keys for Brazilian fiddler crabs elaborated by Crane (1975), Melo (1996) and Bezerra (2012), the number of misidentifications found in various scientific collections is remarkable (see these three authors). The aim of this paper is to present an illustrated identification key for the species of fiddler crabs occurring at the Brazilian Atlantic coast in order to aid undergraduate students and early researchers.

## Materials and methods

Ten species of fiddler crabs recorded along Brazilian coast were analyzed, each one represented by numerous individuals. Most of them (seven species) were collected in various habitats of Guaratuba Bay, municipality of Guaratuba, southern Brazil: *Minuca burgersi* (Holthuis, 1967), *M. mordax* (Smith, 1870), *M. rapax* (Smith, 1870), *Leptuca leptodactyla* (Rathbun, 1898), *L. thayeri* (Rathbun, 1900), *L. uruguayensis* (Nobili, 1901), and *Uca maracoani* (Latreille, 1802–1803). These crabs were deposited in the Natural Museum of Natural History of Capão da Imbuia located in Curitiba, Paraná State, southern Brazil. The remaining three species were obtained from other locations in Brazil: *L. cumulanta* (Crane, 1943) from Natal (Rio Grande do Norte state, northeastern Brazil), *M. vocator* (Herbst, 1804) from Cananeia (São Paulo state, southeastern Brazil) and *M. victoriana* (von Hagen, 1987) from Guarapari (Espírito Santo state, southeastern Brazil); the specimens of the latter species were deposited at the Museum of Zoology of University of São Paulo.

The illustrated key was elaborated as simple as possible, and only clearly visible characters were selected. The key is exclusively based on adult male individuals, as they are provided with the diagnostic characters of the species. As fiddler crabs have gregarious habits, male individuals are hardly absent in the populations.

Line drawings were prepared using a drawing tube attached to a stereoscopic microscope. The systematic nomenclature was based on Shih et al. (2016) and morphological terminology follows Crane (1975). Additional practical characters were added in the item “Recognition characters”. Information on geographical distribution of the species (Table 1) was based on Crane (1975), Mendes and Couto (2001), Koch et al. (2005), Baptista and Calado (2007), Bezerra (2012), Thurman et al. (2013), Pillon (2014), Martins (2018), and Silva (2019). Furthermore, the occurrence of *Uca maracoani* at coast of Santa Catarina state was based on the observation of S.B. Martins (pers. comm.).

The pile, an important morphological feature, is a wooly pubescence on the surface of carapace and ambulatory legs of some species. It is a somewhat difficult to be recognized by beginners, especially in crabs that were preserved in liquids. Drying the specimens in the open air is a practical clue to facilitate the visualization: the piles appear as clear and rough patches on the darkened surface of the carapace or ambulatory legs. It is highly recommended that beginners learn to distinguish these piles, since some morphologically similar species can be easily identified by observing the distribution of these patches.

**Table 1.** Geographic distribution of the fiddler crab species along the Atlantic coast of Brazil. The states were organized by increasing southern latitudes (from left to right), except Amapá that is located northern to Equator. Abbreviations: AL = Alagoas, AP = Amapá, BA = Bahia, CE = Ceará, ES = Espírito Santo, MA = Maranhão, PA = Pará, PB = Paraíba, PE = Pernambuco, PI = Piauí, PR = Paraná, RJ = Rio de Janeiro, RN = Rio Grande do Norte, RS = Rio Grande do Sul, SC = Santa Catarina, SE = Sergipe, SP = . Black circle = occurrence recorded; white circle = probable occurrence but not officially recorded; black square = presence of mangrove.

**States of Brazil**	**AP**	**Equator**	**PA**	**MA**	**PI**	**CE**	**RN**	**PB**	**PE**	**AL**	**SE**	**BA**	**ES**	**RJ**	**Tropic of Capricorn**	**SP**	**PR**	**SC**	**RS**
Mangrove	■		■	■	■	■	■	■	■	■	■	■	■	■		■	■	■	
*Minuca mordax*	•		•	•	○	•	•	○	•	•	•	•	•	•		•	•	•	•
*Minuca rapax*	•		•	•	•	•	•	•	•	•	•	•	•	•		•	•	•	
*Uca maracoani*	•		•	•	○	•	•	•	•	•	•	•	•	•		•	•	•	
*Leptuca leptodactyla*			•	•	•	•	•	•	•	•	•	•	•	•		•	•	•	
*Leptuca thayeri*			•	•	○	•	•	•	•	•	•	•	•	•		•	•	•	
*Minuca burgersi*			•	•	○	•	•	•	•	•	•	•	•	•		•	•	•	
*Minuca vocator*	•		•	•	○	•	○	•	•	•	•	•	○	•		•			
*Leptuca cumulanta*	•		•	•	○	•	•	•	•	•	•	•	○	•					
*Minuca victoriana*						•			•			•	•	•		•			
*Leptuca uruguayensis*														•		•	•	•	•

## Key to the species of fiddler crabs from Brazil

**Table:** 

1	Narrow front, width less than or equal to 15% of front–orbital breadth (Fig. 1) (big and medium-sized crabs)	**2**
–	Wide and triangular front, width more than 15% of front–orbital breadth (Fig. 1B) (medium-sized and small crabs)	**3**
2	Spatulate front (Fig. 1A), width equal to or less than 4% of front–orbital breadth; carapace with bare dorsal surface (Fig. 2A); male major claw with flat fingers like two blades (Fig. 2B) (big crabs, adults can reach up to 45.0 mm carapace width CW)	***Uca maracoani***
–	Triangular front, base ca. 15% of front–orbital breadth; patches of pile (= woolly pubescence, easily detached) on dorsal surface of carapace (Fig. 2C) and on ambulatory legs; major claw of males with cylindrical fingers (Fig. 2D) (medium-sized crabs, adult males can reach up to 28.4 mm carapace width)	***Leptuca thayeri***
3	Carapace provided with major and minor pairs of postero–lateral striae (Figs 3A, 5B, 6A, 8A, C) (medium-sized crabs, adult males with maximum CW 19.0–29.0 mm)	**4**
–	Carapace provided with a single pair of postero–lateral striae (Figs 10A, 11A, C) (small crabs, adult males with maximum of 15.0 mm CW)	**8**
4	Exuberant pile on the dorsal surface of the carapace forming a typical pattern (Fig. 3A) and on all segments of ambulatory legs except dactyl (Fig. 3B)	***Minuca vocator***
–	Carapace with discrete pile or without any, but segments of ambulatory legs (at least from 1^st^ to 3^rd^ pairs) with pile (Figs 4A, B)	**5**
5	Male major chela provided with a short and straight depression filled with pile at the base of pollex (Fig. 5C); discrete pile on carpus and manus (adult male CW maximum 19.1 mm)	***Minuca victoriana***
–	Male major chela without any depression at the base of pollex (Fig. 6B) (adult male CW up to 29.0 mm)	**6**
6	Pile on dorsal surface of carpus and merus and around the entire surface of manus of ambulatory legs (1^st^ to 3^rd^ pairs) (Fig. 4A)	***Minuca mordax***
–	Pile limited to dorsal surface of carpus and manus of ambulatory legs (1^st^ to 3^rd^ pairs); merus without pile (Fig. 4B)	**7**
7	Ambulatory legs with wide merus and dorsal margin convex; merus of last pair of ambulatory legs more than two times wider than respective carpus in its maximum breadth (Fig. 7A)	***Minuca rapax***
–	Ambulatory legs with narrow merus and dorsal margin almost straight; merus of last pair of ambulatory legs less than two times wider than respective carpus in its maximum breadth (Fig. 7B)	***Minuca burgersi***
8	Abdomen with middle somites fused (Fig. 9A)	**9**
–	Abdomen with all somites distinct (Fig. 9B)	***Leptuca cumulanta*** (Fig. 10A, B)
9	Manus of major claw provided with a long groove on dorsal surface following its margin, mostly filled with dirt (Fig. 11B); length of major claw fingers ca. 1.3 times longer than manus	***Leptuca uruguayensis***
–	Manus of major claw without any groove on dorsal surface (Fig. 11D); length of major claw fingers at least 1.6 times longer than manus	***Leptuca leptodactyla***

**Figure 1.**
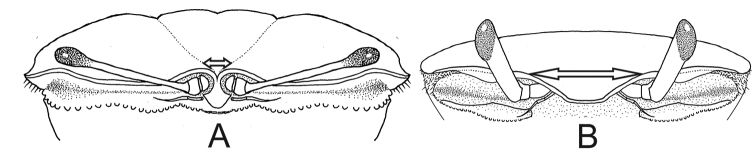
Carapace, frontal view. **A***Uca maracoani*: spatulate and narrow front (seta) **B***Minuca burgersi*: triangulate and wide front (seta).

**Figure 2.**
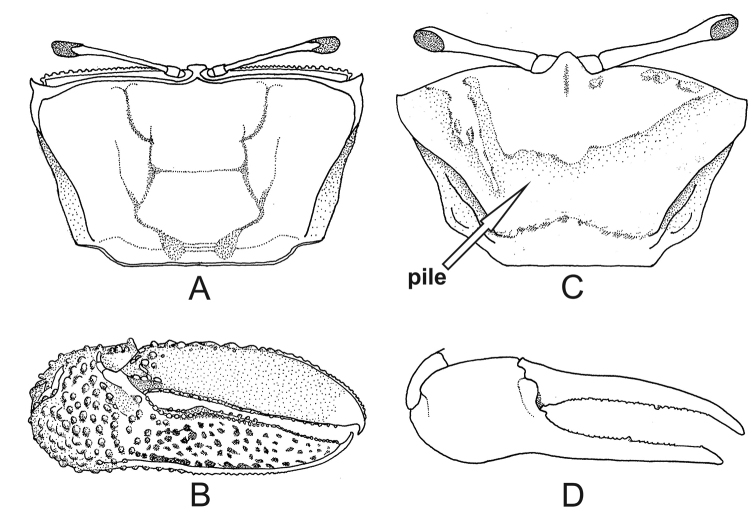
*Uca maracoani*. **A** carapace with bare surface, dorsal view **B** male major claw with flat fingers, frontal view. *Leptuca thayeri***C** carapace mostly covered with pile (seta) **D** male major claw with cylindrical fingers, frontal view.

**Figure 3.**
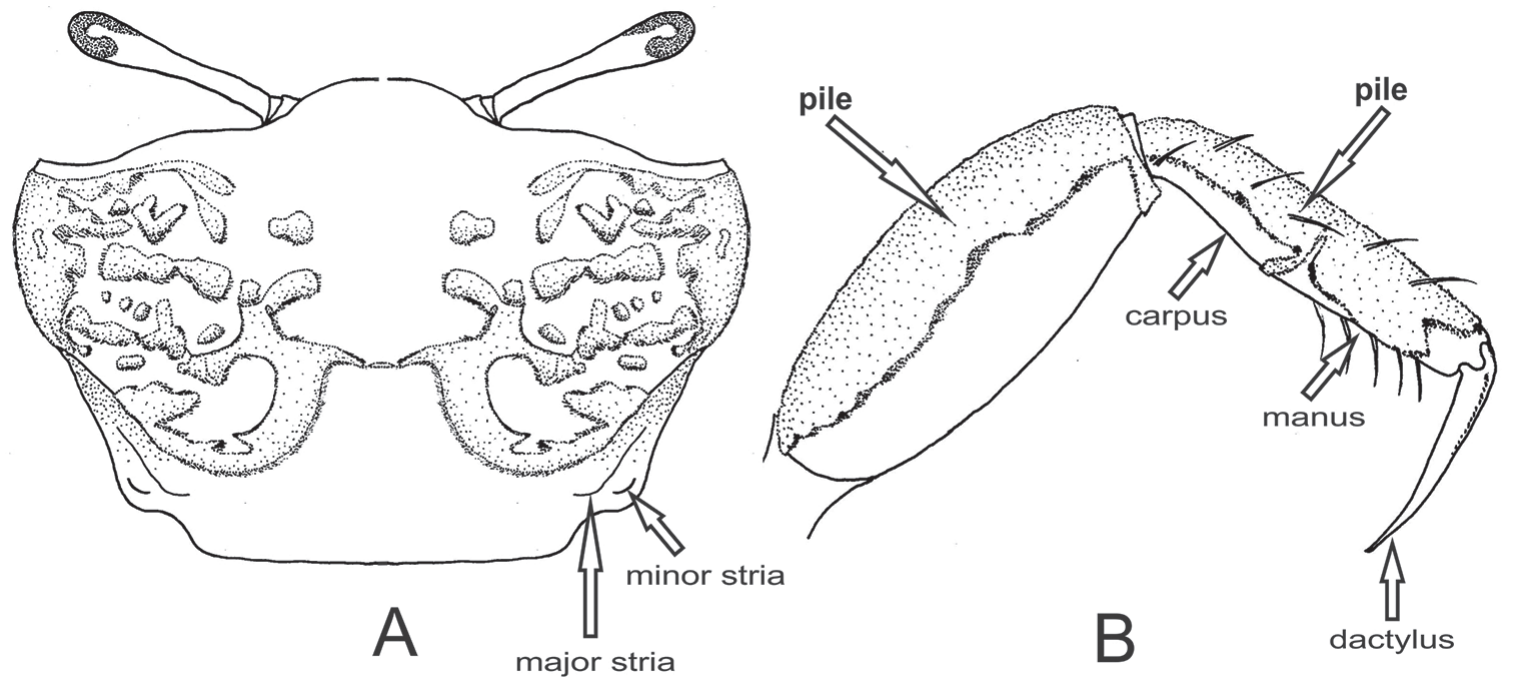
*Minuca vocator*. **A** carapace with pile forming a typical pattern, dorsal view **B** third ambulatory leg with pile on dorsal surface of all segments except dactylus (setae), posterior view.

**Figure 4.**
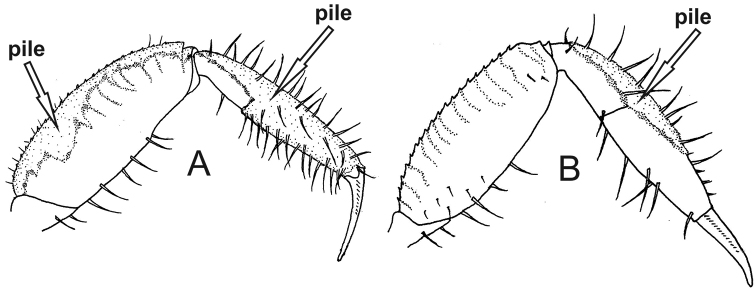
Third ambulatory leg, posterior view. **A***Minuca mordax* with pile on dorsal surface of merus (seta) and carpus and all around surfaces of manus (seta) **B***Minuca burgersi* with pile limited to dorsal surface of carpus and manus (seta).

**Figure 5.**
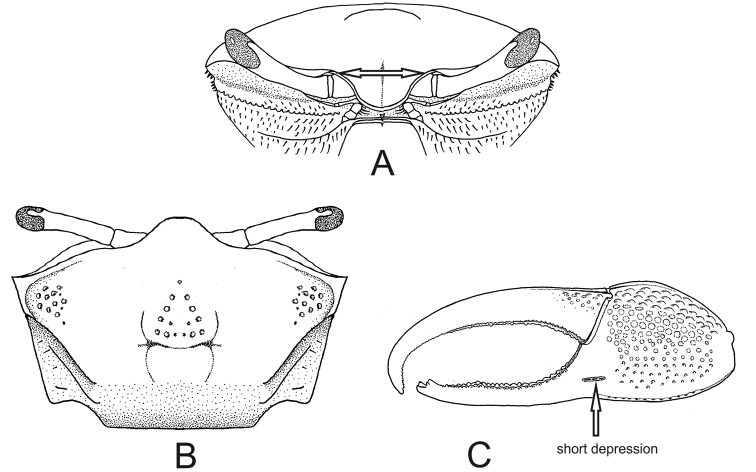
*Minuca victoriana*. **A** carapace with moderately large front, frontal view **B** carapace with two pairs of postero-lateral striae, dorsal view **C** male major claw with a short depression at the base of pollex (seta), frontal view.

**Figure 6.**
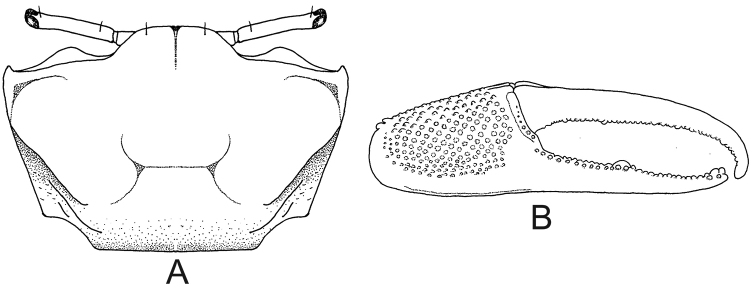
*Minuca mordax*. **A** carapace without pile and with two pairs of postero-lateral striae, dorsal view **B** male major claw, frontal view.

**Figure 7.**
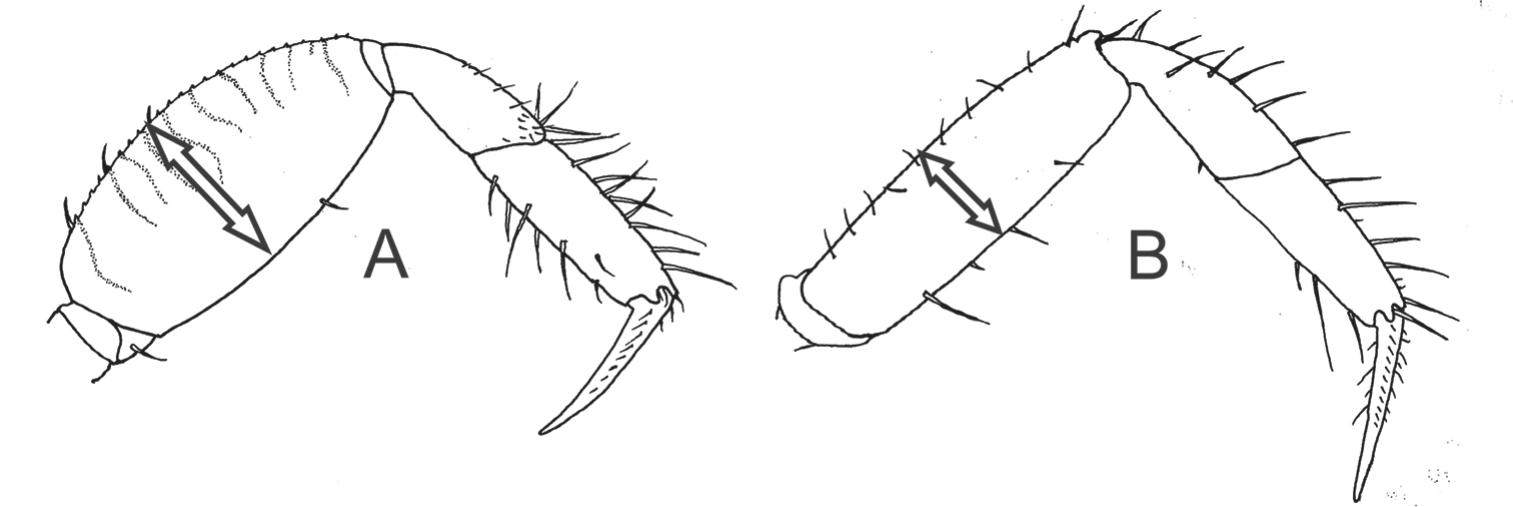
Last ambulatory legs, posterior view. **A***Minuca rapax*, wide merus (seta). **B***Minuca burgersi*, narrow merus (seta).

**Figure 8.**
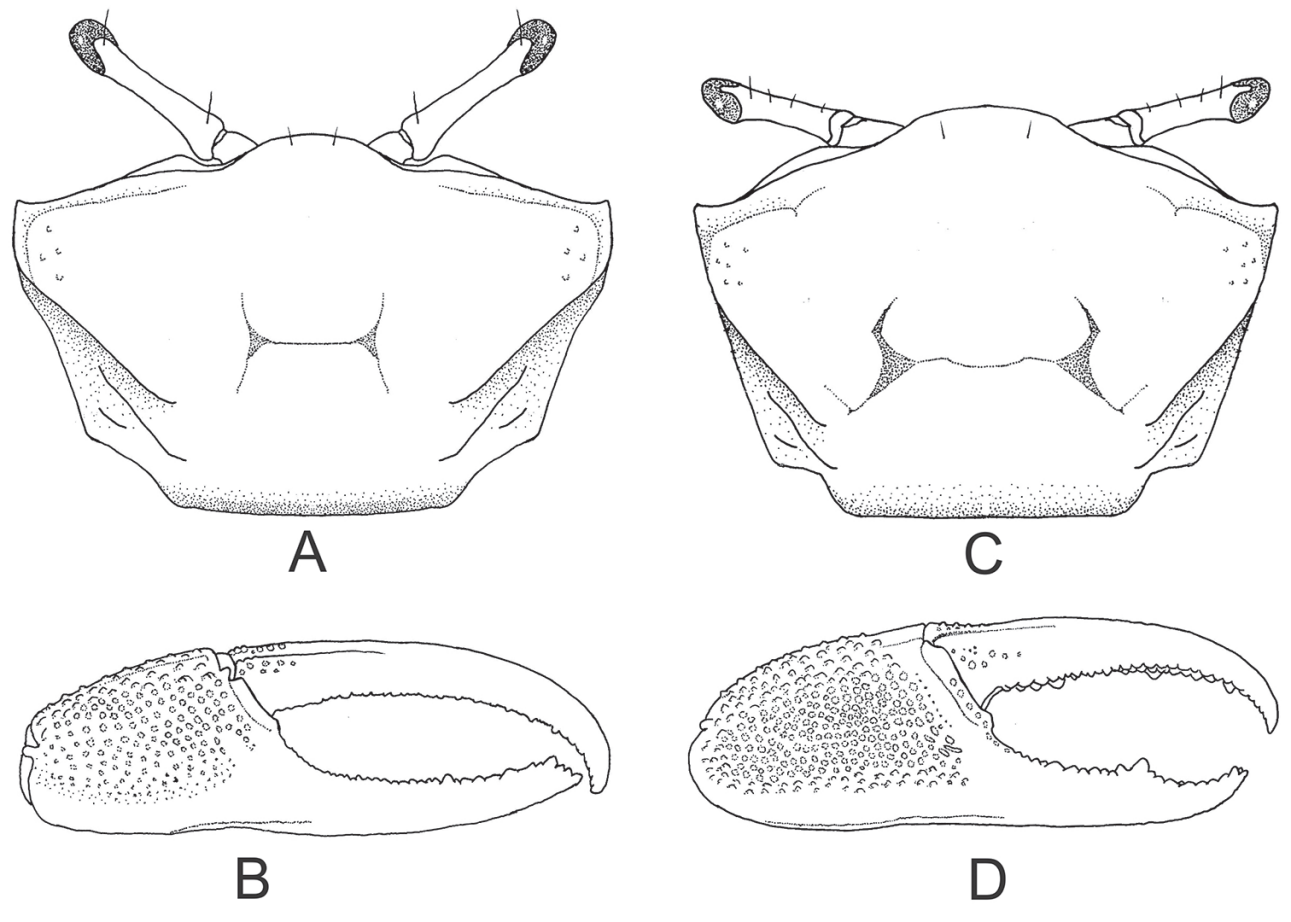
*Minuca rapax*. **A** carapace with two pairs of postero–lateral striae, dorsal view **B** male major claw, frontal view. *Minuca burgersi***C** Carapace with two pairs of postero-lateral striae, dorsal view **D** male major claw, frontal view.

**Figure 9.**
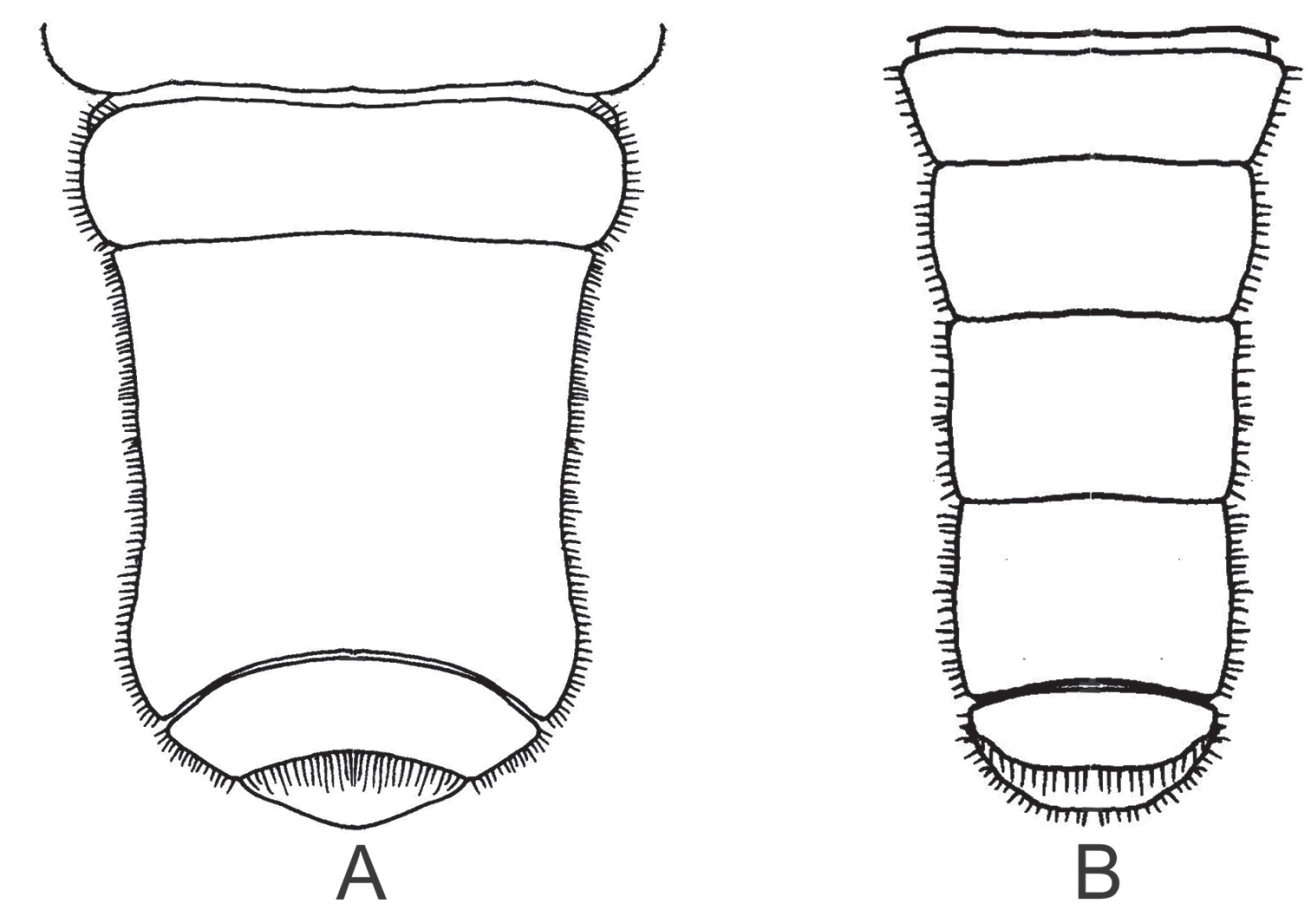
Abdomen of male, ventral view. **A***Leptuca leptodactyla*, middle somites fused **B***Leptuca cumulanta*, all somites distinct.

**Figure 10.**
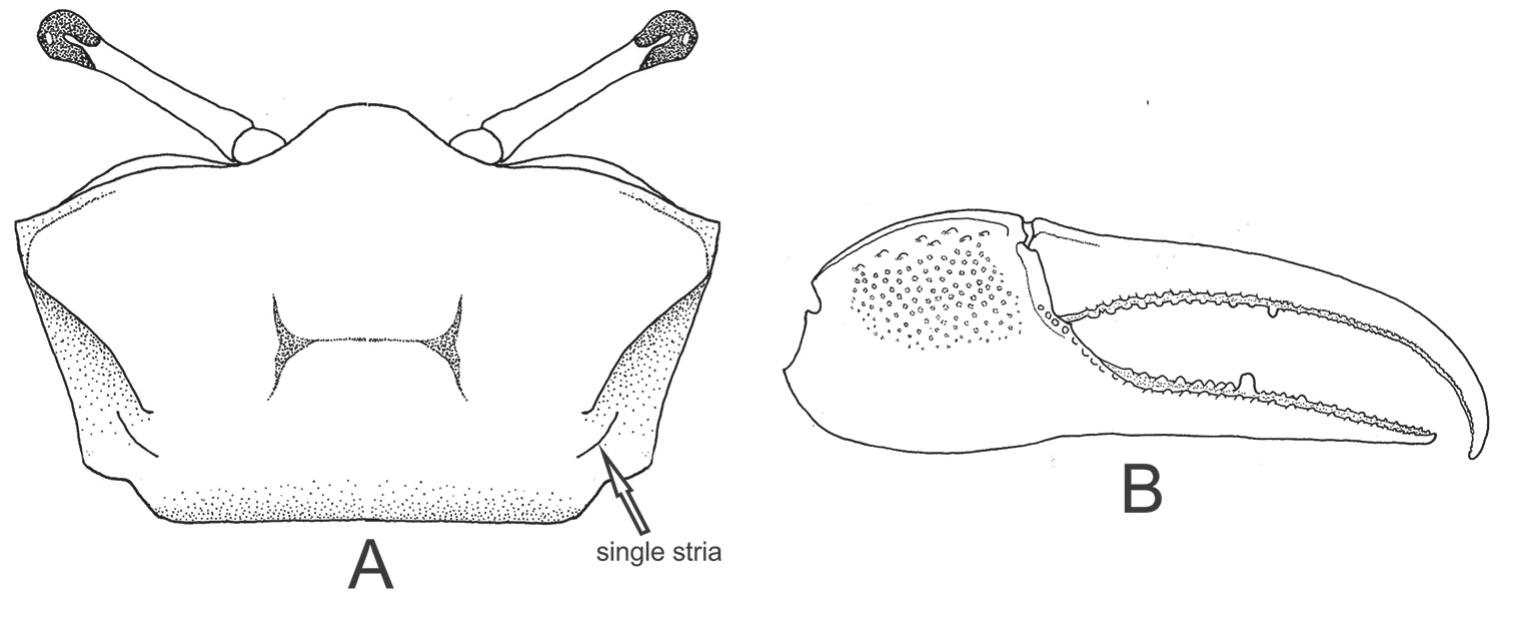
*Leptuca cumulanta*. **A** carapace with a pair of postero–lateral striae (seta), dorsal view **B** male major claw, frontal view.

**Figure 11.**
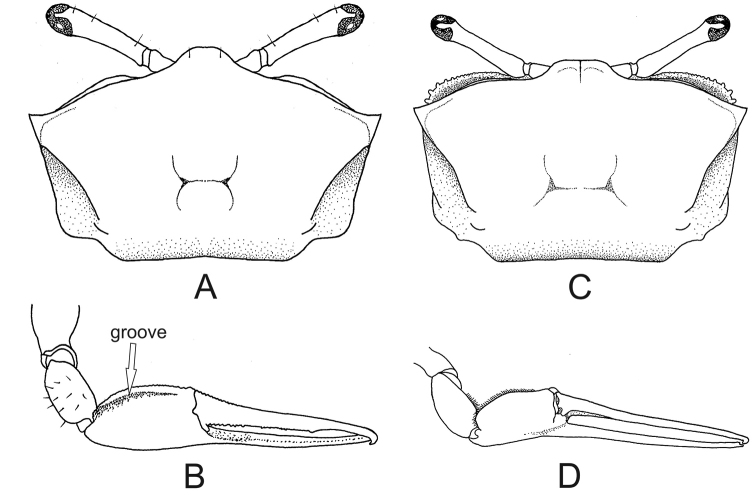
*Leptuca uruguayensis*. **A** carapace with a pair of postero–lateral striae, dorsal view **B** male major claw with dorsal groove (seta), dorso-frontal view. *Leptuca leptodactyla***C** carapace with a pair of postero-lateral striae, dorsal view **D** male major claw without dorsal groove, dorso-frontal view.

## Taxonomy

### Subfamily Gelasiminae

#### Minuca burgersi

Taxon classificationAnimaliaDecapodaOcypodidae

(Holthuis, 1967)

BE75529E-69B2-5CED-A6F4-0CAE0D8874F4

Figures 1B , 4B , 7B , 8C, D

##### Recognition characters.

Carapace pentagonal moderately arched in the anteroposterior direction and dorsal surface without pile (Fig. 8C). Dorso-lateral margins well-marked and converging posteriorly; major and minor pairs of postero-lateral striae clearly visible (Fig. 8C). Front triangular and very wide making up from 36% to 41% of the front-orbital breadth (Fig. 1B). Male major claw manus covered by small tubercles and provided with a strong groove (sometimes filled with dirt) on dorsal surface following the dorsal margin; fingers thick and slightly flattened; dactyl little longer than manus; pollex and dactyl curved forming a large gap (Fig. 8D). First three ambulatory legs with pile (= woolly pubescence) limited to dorsal surface of carpus and manus (Fig. 4B, setae), absent in ventral margin; all ambulatory legs with narrow merus and dorsal margin almost strait; last pair of ambulatory legs without piles and merus less than two 1.5 times wider than respective carpus in its maximum breadth (Fig. 7B). Male abdominal segments never fused. Medium-sized species and one of the smallest in the genus; males’ carapace width (CW) up to 19.0 mm in a population from Fortaleza, CE, Brazil (Crane 1975).

##### Biological notes.

The species reproduces year-round in the population of Ubatuba, southeastern Brazil (Benetti et al. 2007). It occurs in oligohaline and mesohaline areas and on sandy substrate although in low densities (Masunari 2006; Thurman et al. 2013).

##### Remarks.

The species is morphologically close to its congeners *M. rapax* and *M. mordax*; the distinguishing characters among these species are treated in the subsequent items.

### Subfamily Gelasiminae

#### Minuca mordax

Taxon classificationAnimaliaDecapodaOcypodidae

(Smith, 1870)

17A58FB6-31DE-5A52-B527-D6576AB76BA2

Figures 4A , 6A, B

##### Recognition characters.

Carapace pentagonal moderately arched and with naked dorsal surface, without pile (Fig. 6A). Dorso-lateral margins well marked and strongly convergent posteriorly; major and minor pairs of postero-lateral striae clearly visible (Fig. 6A). Front triangular and very wide making up between 34% to 38% of the front-orbital breadth. Male major claw with manus covered by tubercles and with strong groove on dorsal surface; fingers thick and slightly flattened; dactyl ca. 1.5 times the manus length; pollex almost straight with tip curved upwards; dactyl strongly arched ending in a curved downward tip; fingers form a wide gap (Fig. 6B). Ambulatory legs with narrow merus and dorsal margin almost strait; 1^st^ to 3^rd^ambulatory legs with pile limited to dorsal surface of merus (weakly) and carpus (strongly), but all around the manus (strongly) (Fig. 4A); last pair with scant pile on merus, carpus and manus. Male abdominal segments never fused. Medium-sized crab: males’ CW up to 26.1 mm in a population from mangrove of Itajaí River, southern Brazil (Scalco et al. 2016).

##### Biological notes.

Ovigerous females were collected inside burrows that were ornamented with poorly structured chimney at Guaratuba Bay, southern Brazil, during a warm month (November) (Martins et al. 2016). The duration of the reproductive period of this species, however, is still unknown. The species dispersal is larval retention type: after larval period in the pelagic environment of the bay, megalopae return to terrestrial areas, by colonizing mats of red algae that grow on humid substrates. Early juveniles seek shelter among entangled thalli of these red algae, and after molting they migrate to soft muddy substrate (S.B. Martins, pers. comm.). Adults live on consolidated sandy banks of rivers flowing into Guaratuba Bay, forming large populations (Masunari 2006).

##### Remarks.

The most conspicuous morphological character of *M. mordax* is the presence of piles covering the entire surface (dorsal, lateral and ventral) of the manus of 1^st^ to 3^rd^ ambulatory legs. This feature allows to easily distinguish *M. mordax* from two other closely related species, *M. burgersi* and *M. rapax*. As no piles are present on carapace of these three species, they hardly will be confused with *Leptuca thayeri* or *Minuca vocator*. In the field, these species can also be distinguished by its respective habitat: while *M. mordax* is mostly found in freshwater or oligohaline areas such as river banks and tidal flats near river mouth, *M. rapax* and *M. burgersi* are mainly found in mesohaline tidal flats, often in co-occurrence.

### Subfamily Gelasiminae

#### Minuca rapax

Taxon classificationAnimaliaDecapodaOcypodidae

(Smith, 1870)

818600D9-ABEB-5A71-99D8-D469BF6BB1D3

Figures 7A , 8A, B

##### Recognition characters.

Carapace pentagonal moderately arched and provided with small and scarce tubercles in the antero-lateral corner (Fig. 8A); some individuals have pile on H-form depression. Dorso-lateral margins well marked and strongly convergent posteriorly (more pronounced in males); major and minor pairs of postero-lateral striae clearly visible (Fig. 8A). Front triangular and very wide making up 30% to 36% of the front-orbital breadth. Male major claw with manus covered with tubercles and provided with strong groove dorsally; fingers thick and slightly flattened; dactyl ca. 1.5 times longer than manus; pollex and dactyl strongly curved forming a large gap (Fig. 8B). Pile limited the dorsal surface of carpus and manus in the first three ambulatory legs; these legs with enlarged merus (especially the 2^nd^ and 3^rd^), dorsal margin convex and dorsal surface with striated ornaments; last leg without piles and merus more than two times wider the respective carpus in their maximum breadth (Fig. 7A, seta). Male abdominal segments never fused. Medium-sized crabs, male CW up to 28.3 mm and female up to 27.3 mm in a population from Itamambuca mangrove, Ubatuba, southeastern Brazil (Castiglioni and Negreiros-Fransozo 2004).

##### Biological notes.

The species reproduces year-round in the populations from northern and southeastern Brazil (Koch et al. 2005, Castiglioni and Negreiros-Fransozo 2006; Costa and Soares-Gomes 2009). It prefers mesohaline to euhaline areas but it can be found in a wide range of salinities, from oligohaline to euhaline; the preferred substrate is firm sandy to silty clay with humus or clayed silt (Thurman et al. 2013).

##### Remarks.

Morphologically very similar to *M. burgersi* and *M. mordax*. *Minuca rapax* can be distinguished from *M. mordax* in not having a pile around the entire surface of manus of 1^st^ to 3^rd^ ambulatory legs. The distinction between *M. rapax* and *M. burgersi*, however, requires an extra attention: both species have piles limited to the dorsal surface of carpus and manus of 1^st^ to 3^rd^ ambulatory legs. The easiest way to distinguish these two species is to compare the last ambulatory leg: while *M. rapax* has a wide merus with convex dorsal margin (Fig. 7A), that of *M. burgersi* is narrow and its margins are almost parallel (Fig. 7B).

### Subfamily Gelasiminae

#### Minuca victoriana

Taxon classificationAnimaliaDecapodaOcypodidae

(von Hagen, 1987)

D96748F5-438C-549B-BA8B-8EB93D9CE32F

Figure 5A–C

##### Recognition characters.

Carapace pentagonal moderately arched and provided with few tubercles on the surface of antero-lateral corners and on mesogastric area (Fig. 5A). Dorso-lateral margins well marked and strongly convergent posteriorly; major and minor pairs of postero-lateral striae clearly visible (Fig. 5B). The discrete pile on the carapace described by von Hagen (1987) was not observed in the specimens examined in the present study. Front triangular and moderately large making up ca. 22 % of front-orbital breath (Fig. 5A, seta). Male major claw with manus covered with tubercles and provided with strong groove on dorsal margin filled with pile; fingers thick and slightly flattened; dactyl ca. 1.8 times longer than manus; pollex and dactyl strongly curved forming a large gap; a short and straight depression filled with pile at the base of pollex (Fig. 5C, seta). Scant pile on dorsal surface of carpus and manus of the first three pair of ambulatory legs. Male abdomen somites not fused. Medium-sized species and one of the smallest in the genus; males’ carapace width (CW) up to 19.1 mm in a population from Vitória, Espírito Santo state, southeastern Brazil (von Hagen 1987).

##### Biological notes.

Although with a wide geographical distribution, the species forms sparse populations constituted by small individuals in impacted mangroves of southeastern Brazil (Bedê et al. 2008). In tropical mangroves, these crabs form relatively dense populations, reaching larger CW than in southern population and preferring muddy substrates. The recruitment of juveniles occurs continuously; however, the reproductive period of the species is still unknown (Castiglioni et al. 2010).

##### Remarks.

The easiest way to recognize this species is to examine the presence of a short and straight depression filled with a pile at the pollex base in the male major claw (Fig. 5C, seta). This character is unique among Brazilian fiddler crabs. Otherwise, the general shape of carapace of *M. victoriana* is similar to *M. rapax*, *M. burgersi* and *M. mordax*.

### Subfamily Gelasiminae

#### Minuca vocator

Taxon classificationAnimaliaDecapodaOcypodidae

(Herbst, 1804)

052BCA91-C9E4-5E05-9DEE-3EAFBD45B52B

Figure 3A, B

##### Recognition characters.

Carapace pentagonal moderately arched; profuse pile on dorsal surface forming a characteristic pattern mostly on hepatic and branchial regions (Fig. 3A). Dorso-lateral margins well marked even covered by pile, and strongly convergent posteriorly; major and minor pairs of postero-lateral striae clearly visible (Fig. 3A, setae). Front triangular and very wide measuring from 36% to 38% of the front-orbital breadth. Male major claw with manus covered with small tubercles dorsally and frontally and provided with a strong groove on dorsal margin usually filled with dirt; fingers thick and slightly flattened, and a little longer than manus; pollex and dactyl slightly curved forming a gap as wide as the fingers in their base. Exuberant piles on dorsal surface of merus, carpus and manus of all ambulatory legs; these piles can extend to ventral side of manus (Fig. 3B). Male abdomen somites never fused. Medium-sized crabs, males with CW up to 27.0 mm in a population from Itamambuca mangrove, Ubatuba, southeastern Brazil (Colpo and Negreiros-Fransozo 2004).

##### Biological notes.

The species forms one of the densest populations composed by large crabs, and its reproductive period coincides with the rainy period in northern Brazil (Koch et al. 2005). The southeast populations, however, have continuous reproduction (Colpo and Negreiros-Fransozo 2003). On the other hand, in impacted mangroves of southeastern Brazil, populations are not dense and crabs are smaller than in other populations (Bedê et al. 2008). The species prefers muddy substrates (Colpo and Negreiros-Fransozo 2003; Thurman et al. 2013). Large and well-constructed chimneys at the entrance of burrows were observed in a population from Venezuela, but there is no record of this ornamentation in any other population including those from Brazilian coast (Crane 1975).

##### Remarks.

Characteristic pubescence pattern on the carapace and dense piles on dorsal surface of ambulatory legs are the best diagnostic characters for distinguishing it from other *Minuca* species recorded in Brazil. Another Brazilian fiddler crab that has an exuberant pile on the carapace surface is *Leptuca thayeri*, easily distinguishable from *M. vocator* by a very narrow front of the former species (compare Figs 2C and 3A). Although Melo (1996) considered Santa Catarina State as the southernmost limit of the geographic distribution of the species, currently the species has been reported only in the states from Amapá to São Paulo (Table 1).

### Subfamily Gelasiminae

#### Leptuca cumulanta

Taxon classificationAnimaliaDecapodaOcypodidae

(Crane, 1943)

3946E2BD-B95C-5F11-99D5-3BA46903717E

Figures 9B , 10A, B

##### Recognition characters.

Carapace semi-cylindrical, width ca. 1.6 times the length; strongly arched and dorsal surface without pile; lateral margins almost parallel (Fig. 10A). Dorso-lateral margins well marked and strongly converging posteriorly; single pair of postero-lateral striae clearly visible (Fig. 10A, seta). The discrete pile on the carapace described by von Hagen (1987) was not observed in the specimens examined in the present study. Front triangular and moderately wide measuring from 25% to 29% of the front-orbital breadth. Manus of male major claw with dorso-lateral surface covered by tubercles except along the strong dorsal groove (mostly filled with dirt); smooth surface in the submarginal longitudinal area; dorsal surface with sparse tubercles while dorso-lateral one with small and dense tubercles; fingers ca. 1.7 times the manus length; pollex almost straight but dactyl strongly arched ending in a curved downward tip, forming a very wide gap (Fig. 10B). Ambulatory legs without pile. Male abdomen somites distinct (Fig. 9B). Small crabs, males’ CW up to 12.5 mm in a population from Caeté mangrove, Pará state, northern Brazil (Koch et al. 2005).

##### Biological notes.

The species reproduces year-round in populations of northern Brazil and the crabs reach the largest CW among all other populations (Koch et al. 2005). In the impacted mangroves, *L. cumulanta* is the fourth most abundant fiddler crab species, but crabs’ CW is the smallest among these populations (Bedê et al. 2008). The species prefers muddy substrates (Thurman et al. 2013). Hoods at the entrance of male burrows were observed in some populations in Venezuela and Curaçao, but there is no such record from populations of the Brazilian coast (Crane 1975).

##### Remarks.

In sympatric area of Brazilian coast *L. cumulanta* can be confused with *L. leptodactyla* (from Pará to Rio de Janeiro) and *L. uruguayensis* (Rio de Janeiro state): they are similar in size and the major male claw is provided with a very curved dactyl forming a wide gap with the pollex. The best ways to distinguish these three species is described when referring to *L. leptodactyla* and *L. uruguayensis* (see below).

### Subfamily Gelasiminae

#### Leptuca leptodactyla

Taxon classificationAnimaliaDecapodaOcypodidae

(Rathbun, 1898)

6B1C883C-D781-52B9-8FD4-4A28F692B45E

Figures 9A , 11C, D

##### Recognition characters.

Carapace semi-cylindrical, width ca. 1.7 times the length; strongly arched and dorsal surface without any pile or other ornaments; lateral margins almost parallel (Fig. 11C). Front triangular and moderately wide making up 20% to 23% of the front-orbital breadth. Dorso-lateral margins well marked and converging posteriorly; short and single pair of postero-lateral striae clearly visible (Fig. 11C). Male major claw smooth, manus with dorsal margin lined up with minute tubercles; fingers very slender and long, dactylus ca. 1.7 times the manus length (Fig. 11D); pollex almost straight and dactyl strongly arched ending in a curved downward tip; very wide gap between fingers. Ambulatory legs with narrow segments and devoid of pile or other ornaments. Male abdomen with 3^rd^ to 6^th^ somites fused (Fig. 9A). Small crabs: male CW maximum 14.29 mm in a population from Itacuruçá mangrove, Sepetiba Bay, southeastern Brazil (Bedê et al. 2008).

##### Biological notes.

One of the most common fiddler crabs in sandy substrate of estuarine intertidal zone, *L. leptodactyla* reproduces year-round in the population of Ceará state (Bezerra and Matthews-Cascon, 2007), but only in warmer months at the southern coast (Masunari 2012). During the reproductive period (September-March in Guaratuba Bay, Paraná state), males construct a typical hood by piling up sand beside the burrow entrance where they stay for usual waving (Masunari 2012). The species shows strong preference for sandy substrate of polyhaline areas (Masunari 2006), and its young individuals can find shelter in the shadow of cordgrasses (S. Masunari, pers. obs.).

##### Remarks.

*Leptuca leptodactyla* may be confused with *L. cumulanta* at the coast from Pará to Rio de Janeiro states. These two species, however, can be distinguished by features of the male abdomen: the middle somites are fused in the former species (Fig. 9A), while in *L. cumulanta* all somites are distinct (Fig. 9B). Furthermore, *L. leptodactyla* may also be confused with *Leptuca uruguayensis* in the sympatric area (from Rio de Janeiro to Santa Catarina state), especially among juvenile specimens. The male major claw of *L. uruguayensis*, however, is provided with a strong groove parallel to the dorsal margin of the manus (even in juvenile specimens) (Fig. 11B, seta), while in *L. leptodactyla* this groove is absent (Fig. 11D).

### Subfamily Gelasiminae

#### Leptuca thayeri

Taxon classificationAnimaliaDecapodaOcypodidae

(Rathbun, 1900)

173C3EE6-1861-5C35-B966-18FA4D420FE4

Figure 2C, D

##### Recognition characters.

Carapace trapezoidal weakly arched and covered with exuberant pile (pubescence easily detached) (Fig. 2C, seta) and strongly converging posteriorly. Dorso-lateral margins well marked and also strongly converging posteriorly; major and minor pairs of postero-lateral striae clearly visible (Fig. 2C). Front triangular and narrow making up ca. 15% of the front-orbital breadth. Male major claw with manus provided with a strong groove on the dorsal surface; fingers cylindrical and smooth; dactyl almost straight in the proximal two-thirds and curving down toward pollex tip and provided with a short but strong groove on the dorsal surface usually filled with dirt; moderate gap between fingers (Fig. 2D). Ambulatory legs with wide merus ca. 3.3 times the width of proximal end of carpus; posterior surface of all segments (except dactyl) of ambulatory legs covered by pile. Male abdomen segments not fused. Medium-sized species: male CW measures up to 28.4 mm in the population from Formoso River mangrove, Pernambuco state, northeastern Brazil (Farias et al. 2014).

##### Biological notes.

Populations living in the northeastern Brazilian coast reproduce only in the rainy season (Ceará state) (Bezerra and Matthews-Cascon 2007) or continuously (Pernambuco state) (Farias et al. 2014), and those from southeastern Brazil during the warmer months (São Paulo state) (Costa et al. 2006). The species prefers typically muddy mangrove substrates in mesohaline areas, and it is the only fiddler crab in Brazil that forms large populations in shaded areas of the mangrove forest in Guaratuba Bay, southern Brazil (Masunari 2006). Males and females of *L. thayeri* can construct year-round highly structured chimneys around the entrance of the burrows; among the burrow with chimneys recorded in the mangrove of Guaratuba Bay, 53.3 % contained non-ovigerous females, 37.7 % ovigerous females and only 9.0 % males. Furthermore, the chimneys belonging to males had always a larger diameter and were lower than those of females’ (T.F. Moreto, pers. comm.).

##### Remarks.

This species is hardly confused with other species of Brazilian fiddler crabs, as it has a very narrow triangular front (see Fig. 2C). Furthermore, the carapace and the ambulatory legs are heavily covered with pile.

### Subfamily Gelasiminae

#### Leptuca uruguayensis

Taxon classificationAnimaliaDecapodaOcypodidae

(Nobili, 1901)

C47C49CC-36DA-5859-B433-1C1BE2238A90

Figure 11A, B

##### Recognition characters.

Carapace semi-pentagonal strongly arched and dorsal surface without pile or other ornaments (Fig. 11A). Dorso-lateral margins well marked and converging posteriorly; short and single pair of postero-lateral striae clearly visible (Fig. 11A). Front triangular and moderately wide making up from 20.0 % to 23.6% of the front-orbital breadth. Manus of male major claw with dorso-lateral surface covered by small tubercles except along the strong submarginal groove, mostly filled with dirt (Fig. 11B, seta); both edges of dorsal margin armed with lined up by tubercles; dactyl moderately long ca. 1.4 times the manus length; pollex almost straight but dactyl strongly arched ending in a curved downward tip, forming a wide gap. Ambulatory legs with narrow segments and without pile. Male abdomen with 4^th^ to 6^th^somites fused. Small crabs: males with CW up to 12.0 mm in a population from Itacuruçá mangrove, Sepetiba Bay, southeastern Brazil (Bedê et al. 2008). *Leptuca uruguayensis*, however, can attain up to 19.5 mm CW in the population from Solís Grande River, Uruguay (Masunari et al. 2017).

##### Biological notes.

The species reproduces year-round in southeastern (Costa et al. 2006) and southern coast (Martins and Masunari 2013). It tolerates a wide range of salinities and is recorded in sandy substrates with a high degree of organic matter (Masunari 2006). In environments where the mangrove forest is absent (such as in the Uruguayan coast), *L. uruguayensis* occurs in marginal lowlands of rivers that flow into estuaries.

##### Remarks.

*Leptuca uruguayensis* can be confused with *L. cumulanta* and *L. leptodactyla* in Rio de Janeiro state coast (these species are sympatric) due to the small size attained by these three species. The easiest way to separate them is by observing the male abdominal segments: among these three species only *L. cumulanta* has all somites distinct (see Fig. 9B) while the other two species have middle somites fused (Fig. 9A). On the other hand, *L. uruguayensis* can be distinguished from *L. leptodactyla*, by having a deep groove (filled with dirt) on dorsal granulated surface of manus of male major claw (Fig. 11B); in contrast, *L. leptodactyla* has the major claw manus with bare surface (Fig. 11D). Furthermore, the carapace of *L. uruguayensis* is semi-pentagonal with dorso–lateral margins moderately converging posteriorly (Fig. 11A), while *L. leptodactyla* has a cylindrical carapace and dorso-lateral margins weakly converging posteriorly (Fig. 11C).

### Subfamily Ocypodinae

#### Uca maracoani

Taxon classificationAnimaliaDecapodaOcypodidae

(Latreille, 1802–1803)

0BAFB38F-B130-50F8-BFE4-43081E240B4B

Figures 1A , 2A, B

##### Recognition characters.

Carapace trapezoidal moderately arched and naked dorsal surface, without any ornaments. Dorso-lateral margins well marked, long and weakly converging posteriorly; postero-lateral striae absent (Fig. 2A). Front spatulate and very narrow making up ca. 4% of front-orbital breath (Fig. 1A). Male major claw extremely large, with flat fingers and ornamented with tubercles, granules and small patches; narrow gap (Fig. 2B). Ambulatory legs without pile. Male abdomen somites distinct. Large fiddler crab: male CW up to 45.0 mm and female 40.2 mm in Paraty Bay, Rio de Janeiro state, southeastern Brazil (Hirose and Negreiros-Fransozo 2008).

##### Biological notes.

The species reproduces year-round in northern (Azevedo et al. 2016), northeastern (Silva et al. 2016), southeastern (Hirose and Negreiros-Fransozo 2008) and southern (Benedetto and Masunari 2009) regions, but only during the dry season in northern region (Koch et al. 2005). Well-established populations are typically recorded in muddy substrates of polyhaline areas of estuaries, where no other fiddler crab species was seen sharing this space (Masunari 2006). Genetic analysis revealed a lack of discernible genetic subdivision among populations of *Uca maracoani* along Brazilian coast; however, geometric morphometric technique showed statistically significant morphological differentiation that would indicate a strong phenotypic plasticity (Wieman et al. 2014).

##### Remarks.

In the field, these crabs are unmistakable recognizable by the flattened fingers of the male major claw. Furthermore, they are visibly larger than any other Brazilian fiddler crab species.

## Distribution

Most Brazilian fiddler crabs occur along the coastal estuaries in environments closely related to mangroves that are distributed in the country from Amapá state to Laguna do Imaruí in the municipality of Laguna (20°30’S), Santa Catarina state (Vale and Schaeffer-Novelli 2018). Only *Minuca mordax* and *Leptuca uruguayensis* exceed southwards into estuarine areas where mangroves do not grow (Table 1). In Rio Grande do Sul state, where mangroves are absent, *M. mordax* lives on marginal banks of streams (S.B. Martins, pers. comm.), while in Uruguayan estuaries *L. uruguayensis* inhabit stream lowlands (Masunari et al. 2017).

We hypothesized that in Piauí State and others, where records of some common fiddler crab species are missing (Table 1, open circles), future collections will certainly fill the gaps. Only four species occur continuously in all states from Amapá to Santa Catarina (*M. mordax*, *M. rapax*, *Uca maracoani* and *L. thayeri*) while another two species are reported from Pará to Santa Catarina (*L. leptodactyla* and *M. burgersi*). Three species do not follow the entire distribution of mangroves (*M. vocator* from Amapá to São Paulo; *L. cumulanta* from Amapá to Rio de Janeiro, and *L. uruguayensis* from Rio de Janeiro to Rio Grande do Sul). The only remaining species (*M. victoriana*) has a restricted distribution and infrequent occurrence (Table 1).

## Supplementary Material

XML Treatment for Minuca burgersi

XML Treatment for Minuca mordax

XML Treatment for Minuca rapax

XML Treatment for Minuca victoriana

XML Treatment for Minuca vocator

XML Treatment for Leptuca cumulanta

XML Treatment for Leptuca leptodactyla

XML Treatment for Leptuca thayeri

XML Treatment for Leptuca uruguayensis

XML Treatment for Uca maracoani
